# ^1^H NMR-Based Metabolomics Profile of Green and Red *Amaranthus* Grown in Open Field versus Greenhouse Cultivation System

**DOI:** 10.3390/metabo14010021

**Published:** 2023-12-28

**Authors:** Lufuno Ethel Nemadodzi, Gudani Millicent Managa

**Affiliations:** Department of Agriculture and Animal Health, University of South Africa, Johannesburg 1709, South Africa

**Keywords:** NMR, *Amaranthus* species, underutilised vegetables, growth conditions, metabolomic profile, growth promoting metabolites, defence mechanism metabolites, stress regulating metabolites

## Abstract

Traditionally, indigenous African leafy vegetables such as *Amaranthus*, blackjack, jute mallow, cleome monophyla, and spider plants have been conventionally and organically grown as weeds in open fields. However, the lack of land space due to the increase in population has resulted in unconventional, modern, and advanced agricultural farming. The introduction of a greenhouse has recently become the second most popular growing system alongside shade net and glasshouse to increase productivity and meet consumers’ demand. Several studies on *Amaranthus* species have solely focused on physiological parameters and nutritional composition, leaving a huge gap on their metabolomic profile of the leaves which is crucial to comprehend when growing *Amaranthus* species in different cropping systems. Therefore, the study aimed to determine the influence of different cropping systems on the release of metabolites of two commonly consumed *Amaranthus* species in South Africa. H^1^ -Nuclear Magnetic Resonance (NMR) tool was used to profile the untargeted metabolites of green (*Amaranthus graecizans* L.) and red (*Amaranthus cruentus* L.) species. A total of 12 metabolites—trehalose, betaine, glutamine, choline, sucrose, caprate, adenosine, asparagine, carnitine, caffeine, aspartate, and alanine—were detected in green amaranth grown in open fields. Except for caffeine, aspartate, and caprate, which were found in the green amaranth grown in open fields, all the other metabolites were detected in the greenhouse grown once. Interestingly, allantoin, which serves as an allelochemical, was the sole distinct metabolite detected in greenhouse cultivated green amaranth. On the contrary, seven similar metabolites were quantified in red amaranth grown in both open fields and greenhouses, apart from caffeine, which was only detected in greenhouse-cultivated red amaranth.

## 1. Introduction

Amaranth was cultivated by early civilizations over 2000 years ago and continues to be used essentially worldwide [1]. It is a popular traditional leafy vegetable grown in the tropical regions of the world including Africa, India, Bangladesh, Sri Lanka, and the Caribbean, as well as through South-East Asia and Latin America [2]. *Amaranthus* genus belongs to the Amaranthaceae family and consists of more than 60 species [3] with an extensive universal distribution, although most species are found in warm temperature and tropical regions of the world [4]. It is a highly efficient crop that thrives under adverse conditions such as drought, high temperatures, and saline soils [5,6]. According to Trucco and Tranel [7] *Amaranthus* species are grouped into three subgenera. The most economically important is the subgenus *Amaranthus* proper, which includes three species: *Amaranthus hypochondriacus* L., *Amaranthus cruentus* L., and *Amaranthus caudatus* L., all of which are domesticated for grain production.

Grain production of amaranth is versatile and can be used in the preparation of various foods due to its source of bioactive compounds, proteins, and has promising potential in industrial applications such as cosmetics, dyes, and biodegradable plastics [8,9]. *Amaranthus* is considered an important multipurpose plant, and can be exploited in many ways depending on its place of origin. For instance, in South Africa it is grown as a leafy vegetable for consumption and/or fodder to feed livestock animals. Numerous *Amaranthus* species are cultivated as ornamentals and pseudo-cereals with high nutritive value (e.g., amaranth grain) and potherbs [10].

According to Managa and Nemadodzi [11] the high nutritional value of *Amaranthus* leaves, specifically Amaranthus *graecizans* L., has made it the most sought-after vegetable in the rural areas among the poor citizens in the Limpopo province of South Africa. The leaves are usually cooked in supplements with other green leafy vegetables such as mallow jew, pumpkin leaves, blackjack, and nightshade, and consumed with starch made of hard porridge commonly known as “Vhuswa” by the VhaVenda tribe, “Vuswa” by the VaTsonga tribe, and “Bokgobe” by the BaPedi tribe. Furthermore, *Amaranthus* leaves are reported to possess anticancer properties by inhibiting the proliferation of liver, breast, and colon cancer cell lines [12]. Amaranth contains astringent, appetizing, and diuretic properties used in the treatment of diarrhea, dysentery [13] inflammation, gonorrhea, and piles [14]. 

Recently, *Amaranthus* has gained substantial popularity due to its nutritional value in both seeds and leaves, providing high-quality protein, unsaturated oil, and other valuable constituents. As a result, species from this genus have been used throughout the world as oilseed crops for special oil production [15]. On the contrary, in the rural parts of South Africa *Amaranthus* is naturally and organically grown in the open fields for leaf consumption and harvested 5–6 times during the peak season (October–April). The seeds are left to shed on their own and emerge in the next season. However, in recent years, there has been a shift in seed harvest and adaptation of selling amaranth seeds amongst reputable seed companies.

The use of greenhouses to grow high-potential crops such as amaranth is a strategy that has attracted interest in recent years [16]. However, in South Africa, both commercial and smallholder farmers are still sceptical about adapting to growing *Amaranthus* in a greenhouse system. Greenhouse has the potential to intensify agricultural production if appropriate measures are taken to enhance the growth of crops and increase yield/ biomass per unit of cultivated area compared to open field conditions [17]. 

According to Nemadodzi et al. [18] metabolomics has been widely applied in several human and plant studies. However, due to few studies conducted using advanced metabolomic tools on African leafy vegetables such as amaranth, spider plant, jute mallow, and pumpkin leaves, there is little and/or limited scientific literature recorded on their biological constituents. Furthermore, the comparison of metabolomic profiling on open-field cultivation versus greenhouse-grown crops requires detailed and in-depth exploration. Plants are a rich source of metabolites that are used in medicines, food additives, nutraceuticals, flavourings, and other commercial applications [19]. The small molecules, intermediates, and compounds produced during metabolism are known as metabolites. Metabolites serve numerous purposes including fuel, structure, signaling, enzyme stimulation and inhibition, catalytic activity, defence, and ingestion [19]. 

In recent times, there has been a high commercial relevance of the secondary metabolites due to their uncountable health benefits which have accounted for an awaking interest to improve their production in various applications [20]. According to Yeo et al. [21], plant-derived metabolites perform different functions in stress responses such as regulating osmotic pressure within cells, preventing oxidation of cell components, preventing infection, and developing pathogenic microorganisms. These metabolites possess anti-inflammatory, anticancerous, and antidiabetic therapeutic properties, which help in the prevention of various diseases such as cardiovascular, respiratory, gastrointestinal, and neurodegenerative disorders caused by high oxidative stress [19]. According to Zandalinas et al. [22] secondary metabolites significantly improve plant growth and survival under different environmental stresses and thereby act as important primary metabolites.

Different metabolomic tools such as nuclear magnetic resonance (NMR) spectroscopy and liquid chromatography-mass spectrum (LC-MS) analyses are commonly used to determine the metabolite profile of various samples [18]. The advantages of using NMR include its non-destructiveness, unbiasedness, and quantitative, uncomplicated sample preparation, and may be used to study compounds that are difficult to detect using gas chromatography-mass spectrum (GC-MS) or LC-MS. According to Anand et al. [19] NMR frequently necessitates the derivatization of substances like sugars and amines, and is highly reproducible [23]. The primary objective of this study was to determine how different cropping systems impact the release of metabolites required for the effective growth and establishment of *Amaranthus* species.

## 2. Materials and Methods

Open field cropping system: the experiment was conducted in Itsani village, situated about 6 km southwest of Thohoyandou in the Thulamela Local Municipality, Vhembe District, Limpopo Province of South Africa. Itsani village has a Latitude of −22.94786, a Longitude of 30.47276, and an altitude of approximately 1140 m above sea level. During the summer planting period (October to March), temperatures range from 26–32 °C with an average rainfall of ±500 mm annually [11]. 

Greenhouse cropping system: the pot experiment was conducted in the greenhouse with minimum and maximum air temperature ranges of 7.4 and 44.9 °C, situated at the University of South Africa, Florida Science Campus, Rooderpoot (Latitude: −26°9′29.274″; Longitude: 27°55′17.663″). The average relative humidity inside the greenhouse was recorded at 68% [24] during the planting period (October 2021 to February 2022). Plastic pots (18 cm diameter, 14.5 cm height, and 18 cm width) were used in growing seeds. The growth cycle lasted for a period of 3 months in the summer seasons of October to December 2021 for both cropping systems.

### 2.1. Leaf Collection

**Figure 1:**
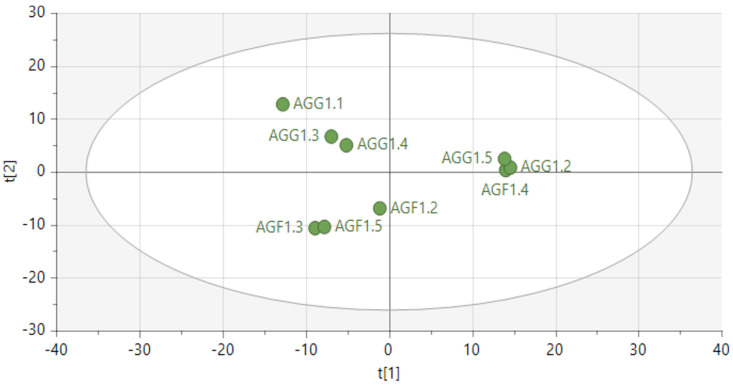
PCA (unsupervised) model scatter plot showing clusters of samples of *A*. *graecizans* L. specie grown in open field herein referred to as Amaranthus green field (AGF) versus cultivated in the greenhouse herein referred to as Amaranthus green greenhouse (AGG).

**Figure 2:**
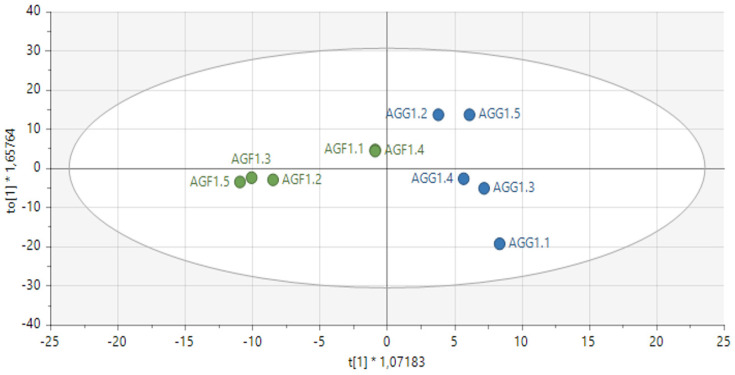
Score plot of OPLS−DA (supervised) model showing the distinct separation between *A*. *graecizans* L. grown in the open field (AGF/green) versus *A*. *graecizans* L. cultivated in the greenhouse (AGG/blue) R2X [1] = 0.222, R2X0 [1] = 0.551 on Hotelling’s T2 (95%). R2X [1] describes the variation in the samples, whilst R2Xo [1] indicates the components of the variable. The sign * at X (vertically) and Y (horizontally) represent the summary of fit tiled on the treatments.

**Figure 3:**
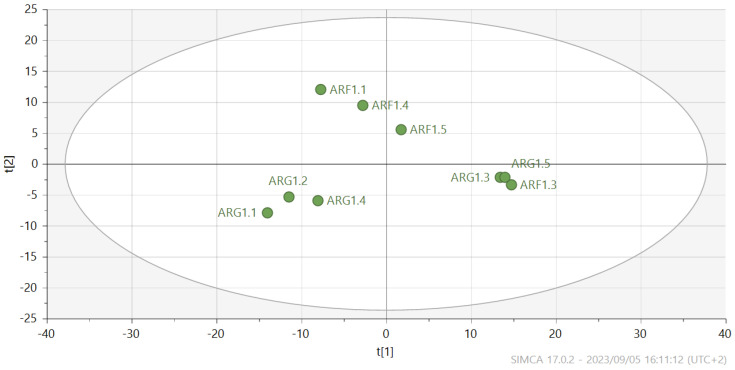
PCA (unsupervised) model scatter plot showing clusters of samples of red amaranth species grown in open field, herein referred to as Amaranthus red field (ARF) versus red amaranth cultivated in the greenhouse, herein referred to as Amaranthus red greenhouse (ARG).

**Figure 4:**
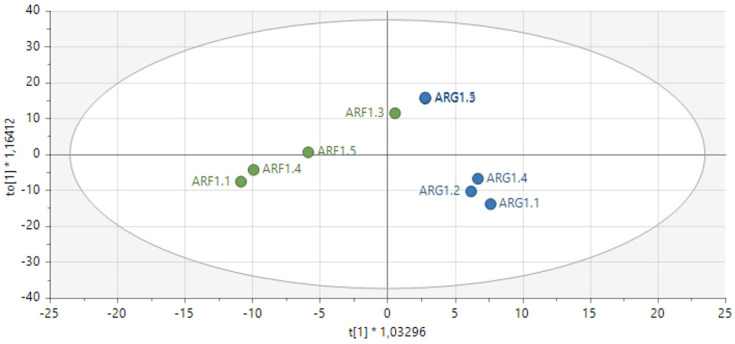
Score-scatter plot using OPLS−DA (supervised) shows a distinct separation of secondary metabolites between Amaranthus red species grown in open field (ARF/green) versus those cultivated in the greenhouse (ARG/blue). R2X [1], whilst R2Xo [1] indicates the components of the variable.

Fresh leaves of green Amaranthus grown in the open field coded as AGF versus green Amaranthus cultivated in the greenhouse coded as AGG (see Figure 1 and Figure 2) and fresh red Amaranthus cultivated in the open field (ARF) and the ones greenhouses cultivated (ARG) as shown in Figure 3 and Figure 4 were harvested in December of 2021, and then stored and dried at 27 °C until use. 

### 2.2. Leaf Sample Preparation

Dried leaves from each cropping system were grounded into fine powder with Russel Hobbs Blender (500 W, Zhongshan, China) to produce homogeneous powdered samples used for NMR metabolomics analysis.

### 2.3. NMR Metabolomics Analysis

For NMR analysis, deuterated methanol (CD_3_OD), KH_2_PO_4_, sodium deuterium oxide (NaOD), trimethylsilyl propionic acid sodium salt (TSP), and deuterium oxide (D_2_O) was supplied by Sigma-Aldrich (Darmstadt, Germany). Buffer was prepared by adding 1.232 g KH_2_PO_4_ to 100 mL of D_2_O with 10 mg TSP (0.1%) added as a reference standard. The pH of the solution was adjusted to pH = 6. The protocol reported in [25] was implemented for the extraction procedures, with a few adjustments. A dried leaf sample of 50 mg was transferred to 2 mL Eppendorf tubes were extracted with 750 µL of deuterated methanol and 750 µL KH_2_PO_4_ buffer in D_2_O (pH 6.0) containing 0.1% TSP. The Eppendorf tubes were vortexed for 1 min at room temperature and ultra-sonicated for 20 min without heating. The solutions were centrifuged for another 15 min at 10,000 rpm to separate the supernatant from the precipitate. The supernatant was transferred to standard 5 mm NMR tubes and subjected to ^1^H NMR analysis.

The ^1^H NMR measurements were performed on a Varian 600 MHz spectrometer (Varian Inc., Palo Alto, CA, USA) with a frequency of 599.74 MHz. The acquisition time of each ^1^H NMR spectrum was 7 min, which consisted of 32 scans with a width of 20 ppm. Gradient shimming was used to improve homogeneity on the magnetic field, and each sample was replicated five times. All spectra were phase-corrected and binned at 0.04 ppm to 10.00 ppm using MestReNova [26] before being statistically analyzed with SIMCA 17.0.2 (Umetrics, Umea, Sweden). Data scaling was done using the Pareto method, an unsupervised principal component analysis (PCA), and a supervised orthogonal partial least squares discriminant analysis (OPLS-DA) model to illustrate the distinctive separation between the two sampling sites where leaves were collected [27,28]. Metabolite annotation, detection, and identification of metabolites were carried out using Chenomx NMR suite 9.0 (Edmonton, AB, Canada) which has specific characteristics, also known as values, for a variety of metabolites [18]. It was used to annotate non-targeted metabolites which were profiled and identified. Chenomx library manager was used to obtain the compounds database and Chenomx compound builder version 11 was used to create ^1^D NMR chemical structures in relation to the cluster/number of peaks, shapes, locations, and/or NMR signal regions. External reference databases such as Chenomx and Human Metabolome Data Base (HMDB), were used to compare and confirm NMR-specific characteristics [18].

## 3. Results

Principal component analysis (PCA), which is the unsupervised model on both *Amaranthus* species, did not show a good separation in the samples from *Amaranthus* species grown in the greenhouses versus those cultivated in the open field (see Figure 1 and Figure 3). Each dot represents the individual sample analysed. Notably, PCA clustered the genus amaranth and could not segregate nor differentiate between the different cropping systems (greenhouse versus field grown). 

However, ^1^H NMR through orthogonal projections to latent structures discriminant analysis (OPLS-DA) showed a distinct separation of the compounds detected on *Amaranthus* species grown in the open field versus greenhouse cultivated as shown in Figure 2 and Figure 4. The predictability of the model showed a perfect fit at 95%, R2X0 (samples variation) = 0.44 and R2X (components of the variable) = 0.229.

Additionally, the NMR spectra clearly showed the trehalose, glutamine, adenosine, alanine, carnitine, caffeine, choline, betaine, aspartate, sucrose, and caprate on green amaranth (see Figure 3a,b) grown in the open field with few of the same metabolites found in a greenhouse cultivated ones.

Table 1 indicates the ^1^H NMR regions and/or peak lists at which secondary metabolites were found in green *Amaranthus* grown in the present study and were compared to the external reference databases such as Chenomx and Human Metabolome Database (HMDB). The carnitine peak at 3.2 ppm had a 0.3898 concentration level, whilst caffeine had different concentration levels with the highest found on 0.1607 at 3.5 signal, 0.1231 at 3.4 signal, the least concentration of 0.919 at 3.9 signal, and 0.1006 at 7.9. On the other hand, betaine’s peak at 3.3 signal had 2.3740, whilst the highest (2.669) concentration was found at 3.9 signal. Trehalose peak/signal at 3.41 ppm had a concentration level of 0.4516 mm. Alanine had 1.0688 concentration level at 1.46 and 1.47 peaks. Furthermore, adenosine and aspartate had 0.3874, while aspartate had concentration levels that ranged from 0.9989 at 2.8 signal to 0.4343 at 3.9 peak. Glutamine showed a concentration level of 2.3401 at 3.86, caprate had 1.0858 at 2.1 signal, choline had 0.4347 at 3.28, sucrose at 1.1976 at 4.2, and asparagine had the highest concentration level at 38.5973 at 6.9 ppm.

**Figure 5:**
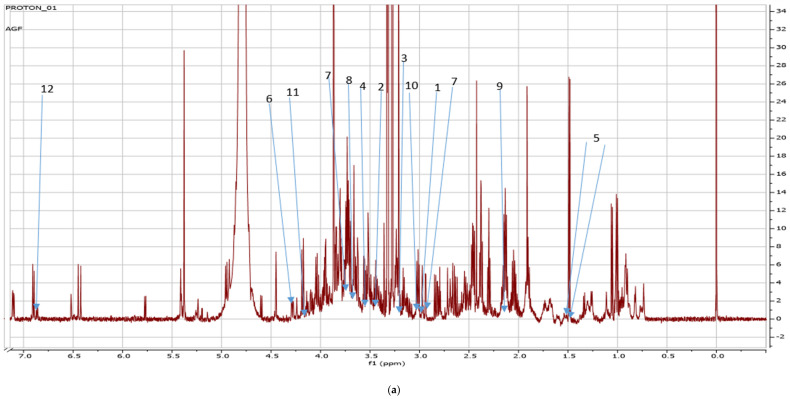

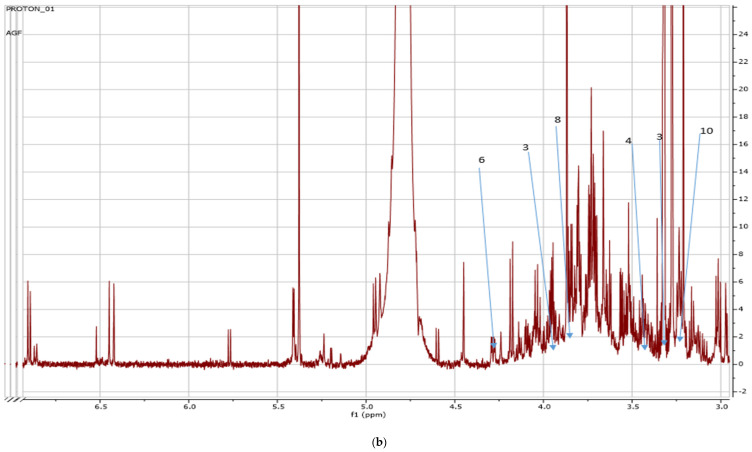
(**a**) NMR spectra showing peaks detected on green *Amaranthus* grown in the open field. Carnitine (1) (3.20 ppm), caffeine (2) (3.49 ppm), betaine (3) (3.30 ppm), trehalose (4) (3.41 ppm), alanine (5) (1.47; 1.49), adenosine (6) (4.31 ppm), aspartate (7) (2.90; 3.90 ppm), glutamine (8) (3.86 ppm), caprate (9) (2.10 ppm), choline (10) (3.28 ppm), sucrose (11) (4.24 ppm), asparagine (12) (6.9 ppm); (**b**) shows the enlargement of overlapping peaks (3, 4, 6, 8, 10) extracted from the original peak (Figure 5a).

**Figure 6:**
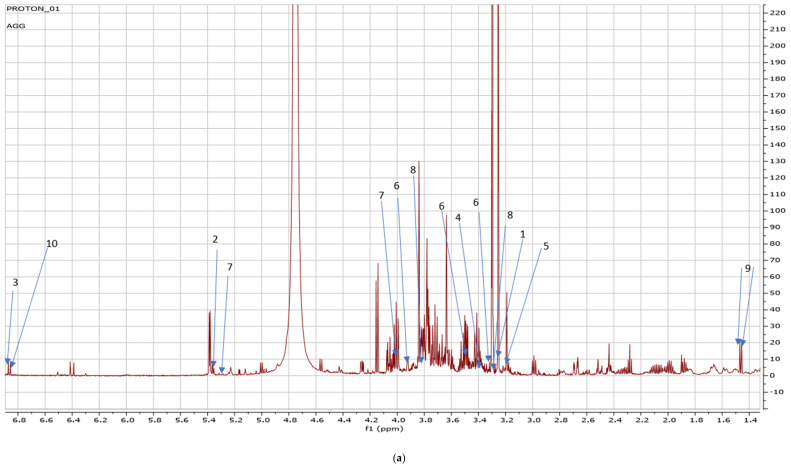

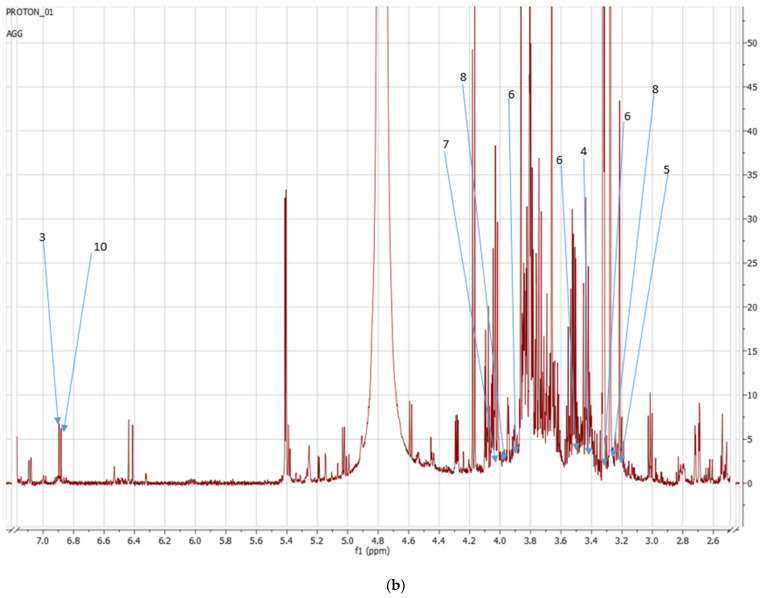
(**a**) The NMR spectra showing peaks detected on green *Amaranthus* cultivated in a greenhouse, carnitine (1) (3.25 ppm), allantoin (2) (5.40 ppm), glutamine (3) (6.89 ppm), trehalose (4) (3.41 ppm), choline (5) (3.35 ppm), caffeine (6) (3.29 ppm; 3.5 ppm; 3. 91 ppm), sucrose (7) (4.0 ppm; 5.38 ppm), betaine (8) (3.25 ppm; 3.89 ppm), alanine (9) (1.45 ppm; 1.46 ppm), asparagine (10) (6.9 ppm); (**b**) indicates the overlapping peaks (3, 4, 5, 6, 7, 8) which were extracted from Figure 4a and enlarged.

**Figure 7:**
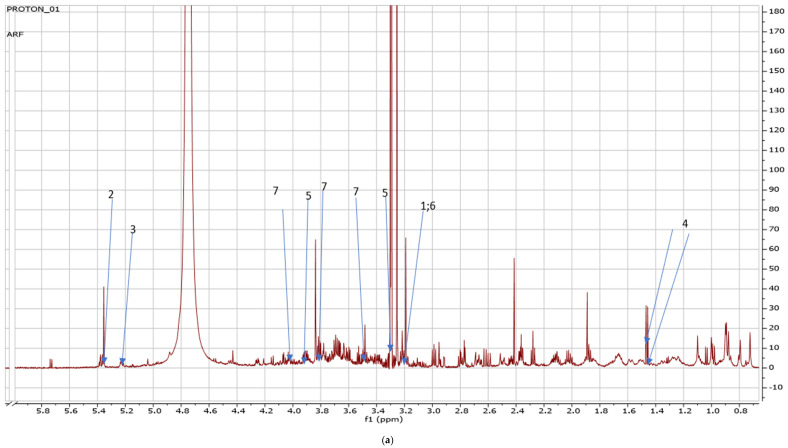

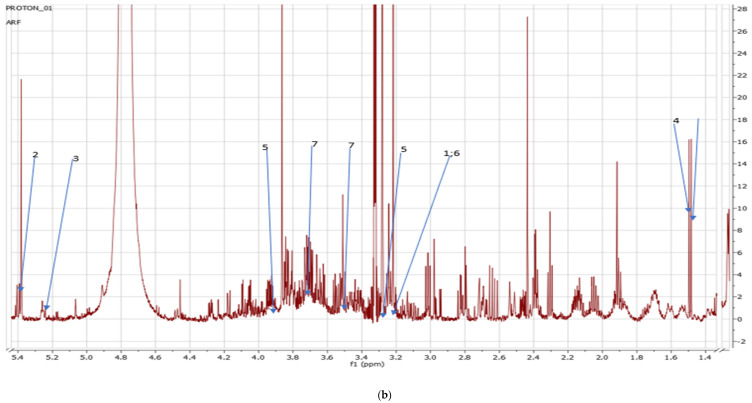
(**a**) The NMR spectra showing peaks detected on red amaranths cultivated in open field, carnitine (1) (3.19 ppm), allantoin (2) (5.35 ppm), trehalose (3) (5. 23 ppm), alanine (4) (1.45 ppm; 1.46 ppm), betaine (5) 3.35 ppm; 3.89 ppm), choline (6) (3.19 ppm), sucrose (7) (3.49 ppm; 3.8 ppm; 4.0 ppm); (**b**) shows enlarged peaks at 1, 2, 3, 4, 5, 6, extracted from Figure 5a.

Due to the number of metabolites detected from each Amaranth species grown in different cropping systems, there was an intense overlap of NMR peaks detected on the spectra labeled Figure 5a, Figure 6b, and Figure 7a. An enlargement of the peaks was performed for clearer detection and easy reference as shown in Figure 5b, Figure 6b, and Figure 7b.

Table 2 indicates the NMR regions and/or signals and/or peak lists at which secondary metabolites were found in green amaranth grown in the present study and were compared to the external reference databases such as Chenomx and Human Metabolome Database (HMDB). Carnitine had a concentration level of 0.3696 at 3.2 signals, allantoin had a 1.2849 concentration level at a 5.4 peak. Glutamine had the second-highest concentration at 121.2804. In addition, a 3.4532 concentration level was found on trehalose at a 3.4 signal whilst the concentration level of choline at 3.25 was 0.4422. Concentration levels of caffeine ranged from 5.7127 at 3.3, 0.4915 at 3.5, 0.2402 at 3.9, and the lowest concentration of 0.0518 at 7.9 signal. On the other hand, sucrose’s concentration levels ranged from 5.3670 to 4.9383 at 4.0 and 5.38 respectively. Interestingly, betaine at 3.3 and 3.9 signals had the same concentration level of 4.6194 whilst alanine at 1.45 and 1.46 had a 0.7722 concentration level. Finally, asparagine at 6.87 signal had the highest concentration level of 275.6270.

The unsupervised model (PCA) used in the current study showed no separation between samples collected from red amaranth grown in open field versus greenhouse cultivated as indicated in Figure 3. Interestingly, PCA only grouped the genus amaranth and could not separate the differences between the two cropping systems used as treatments in the current study.

On the contrary, OPLS−DA revealed a clear difference in the samples (red amaranth) analyzed which were field grown (green) versus greenhouse (blue) grown as shown in Figure 4.

Table 3 shows the metabolites detected in the specific NMR regions which are compared to the external database references. Carnitine had a 0.6029 concentration level at 3.19 signal, allantoin at 5.35 had the highest concentration level of 8.2139 and the concentration level of trehalose was 0.1176 at 5.23. In addition, alanine at 1.45 and 1.46 signals had 0.7942 concentration levels whilst betaine at 3.25 and 3.87 had the same concentration level at 2.2404. Furthermore, choline at the 3.19 signal had a 0.5759 concentration level, and sucrose concentration levels ranged from 1.1082, 1.0688, and 0.5123 at the signals of 3.49, 3.8, and 4.0 respectively.

**Figure 8:**
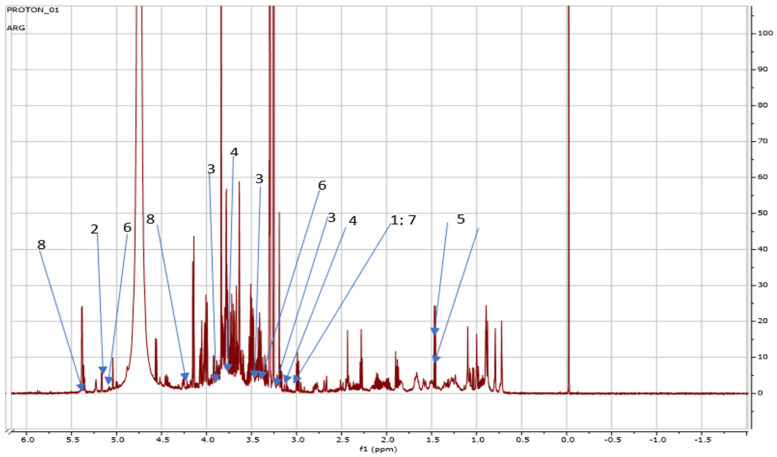
NMR spectra showing peaks detected on red *Amaranthus* grown in a greenhouse, carnitine (1) (3.19 ppm), allantoin (2) (5.35 ppm), caffeine (3), (3.3 ppm; 3. 47 ppm; 3.91 ppm), betaine (4) (3.25 ppm; 3.89 ppm), alanine (5) (1.45 ppm; 1.46 ppm), trehalose (6) (3.46 ppm; 5.16 ppm), choline (7) (3.19 ppm), sucrose (8) (4.21 ppm; 5.38 ppm).

Interestingly, red Amaranthus cultivated in the greenhouse system possessed seven metabolites which were also detected in the red Amaranthus grown in the open field apart from caffeine which also had more peaks compared to other metabolites detected as shown in Figure 8.

Table 4 below indicates the NMR regions and/or peak lists at which secondary metabolites found in red amaranths grown in the present study were compared to the external reference databases such as Chenomx and Human Metabolome Database (HMDB). Carnitine at 3.19 signal had a 0.3952 concentration level, allantoin had 2.3099 at 5.35 whilst caffeine concentration levels ranged from 2.2110 at 3.3 to 0.2618 at 3.47 and 3.91 signals. Notably, both peaks for betaine had the same concentration of 4.0918 whilst alanine at 1.45 and 1.46 signals had a concentration level of 0.6938. On the other hand, trehalose had a concentration level of 1.0251 at 3.46 and 0.4750 at 5.16 signals, and choline at a signal of 3.19 exhibited a concentration level of 0.4428. Finally, sucrose at 5.38 and 4.21 had the same concentration level at 4.0677.

## 4. Discussion

Harvesting of traditional or African indigenous leafy vegetables in rural communities is mostly done at different stages of plant growth, at which regrowth of the new leaves happens after each harvest. It is likely that for some of these indigenous leafy vegetables, there is a preferred stage of plant development when flavour and palatability are favourable for human consumption [31]. In the present study, harvesting occurred based on maturity stage, therefore it is assumed that the metabolites detected could be influenced by the growth stage of the crop. Although *Amaranthus* species are probably the most widely occurring leafy vegetables in South Africa and Africa in general [32], scientific literature on the metabolomic profile of two common *Amaranthus* species (*A*. *cruentus* and *A*. *graecizans*) grown and consumed in South Africa is limited. Most studies on African leafy vegetables are focused on carotenoids, flavonoids, agronomic parameters, and nutritional composition, leaving a significant gap in plant metabolites. Notably, the current study makes it the first research conducted to compare the metabolites released by two aforementioned *Amaranthus* species grown in open field versus greenhouse cropping systems using ^1^H Nuclear Magnetic resonance.

^1^H NMR metabolomic profile through the OPLS-DA model and analysis showed a clear, distinct separation of the *Amaranthus* grown in two different cropping systems. A contribution plot was used to detect and identify the ^1^H NMR peak regions responsible for the clustering and separation of the metabolites. Between the two cropping systems, higher concentrations and intensities of secondary metabolites were found in a green *Amaranthus* species grown in open field. Interestingly, few of the metabolites found in open field-grown amaranth species were also detected in greenhouse-cultivated species.

Trehalose and glutamine detected in this study are proposed to be secondary metabolites responsible for the growth and development of *Amaranthus* species, herein referred to as growth-promoting metabolites. A study by Nemadodzi et al. [18] agrees with our findings on trehalose being the growth-promoting metabolite (GPM) required to sustain the growth of *B*. *africana* trees. Another study by Kumari et al. [33] concurs with the aforementioned points and refers to trehalose as a halotolerant plant growth-promoting rhizobacteria (HT-PGTR), also known for their capability to tolerate and mitigate salinity stress in plants and significantly enhanced germination percentage, germination rate, and index of wheat seeds by 43, 51, and 123%, respectively, as compared to control.

Furthermore, a study on Arabidopsis [34] showed that trehalose was responsible for seed maturation. Eastmond et al. [35] reported that in the absence of functional trehalose, no transition from vegetative generation development occurred, and Arabidopsis plants were found to be retarded in growth with small rosette leaves.

On the contrary, a study by Dijken et al. [36] disagrees with the aforementioned claims and stated that trehalose inhibits growth and is toxic when fed to seedlings of the dodder vine *Cuscuta reflexa*. According to Thierry et al. [37] the negative impact of trehalose is strange, as trehalose is reported as a common sugar that is synthesized in many organisms at high concentrations, playing the role of a carbon source and stress protection compound. Their findings reported that trehalose at 100 mM in half-strength Murashige and Skoog (MS) medium inhibited the growth of Arabidopsis seedlings of all accessions tested. Furthermore, their findings revealed that seedlings germinated on these medium developed short roots less than 3 mm long after 14 days, and the leaf primordia did not extend into leaves compared with normal development on 100 mM sorbitol osmoticum control due to a large accumulation of starch [37].

According to Schluepmann et al. [38], betaine also improves the production of photosynthetic pigments, chlorophyll, and enhances morphological features such as shoot fresh dry weight, leaf length, and number of leaves. Several studies on cotton, [37,38,39,40,41] reported that glycine betaine diminishes deleterious injuries under water deficit conditions and improves growth. The findings by Naidu et al. [40] showed that the application of glycine betaine resulted in a significant increase in shoot dry weight, and reduced stress through several different mechanisms. A study by Hwang et al. [42] reported that asparagine and glutamine are necessary for the production, metabolism, and transportation of nitrogen needed for the growth and development of plants. Luo et al. [43] concurred with the aforementioned point that asparagine and glutamine are the most important forms of N transport from roots to shoots. According to Gaufichon et al. [44], asparagine is found in plants, regulated by light, and is involved in seed development and vegetative growth. Furthermore, asparagine has been reported as one of the most vital metabolites that play a central role in circulating nitrogen and recycling in plant vegetative organs [45].

A study conducted in rice xylem and phloem revealed that glutamine was the most abundant amino acid, followed by asparagine in the transportation of N [46,47]. In a study by Maluleke [48] asparagine content was reported in African horned cucumber fruit grown under different environmental conditions (greenhouse, shade net, and open field). Trehalose, betaine, sucrose, and alanine detected in this study were reported by Nkobole and Prinsloo [49] among other metabolites in the wild and cultivated *A*. *cruentus* and *A*. *hybrids* species.

Plants can modulate their developmental programs through the integration of external abiotic signals [50]. The plant’s life cycle goes through an enormous phase of environmental changes [51]. In response to these changes, plants accumulate trehalose, allantoin, and other consistent compounds to maintain osmotic homeostasis against cold and drought [52]. Several studies reported that the production of secondary metabolites such as trehalose directly modulates plant’s cellular responses through the regulation of salt overly sensitive gene [53] expression of stress-regulating genes and is involved in the alleviation of salt-stress. As reported by Li et al., Sogbohossou, and Achigan-Dako [5,6], *Amaranthus* is a highly efficient crop that thrives under adverse conditions such as drought, high temperatures, and saline soils. In agreement with our findings, Obata et al. [54] reported on trehalose content in the plants grown in both greenhouses and field-grown plants. From the findings of our study, it is proposed that trehalose, betaine, asparagine, and glutamine detected in the leaves of *Amaranthus* spp. serve dual-purpose roles as growth-promoting metabolites (GPM) and growth-regulating metabolites (GRM). A combination of these metabolites influences the effective growth of *Amaranthus* species.

In addition, metabolites such as alanine, carnitine, and caffeine detected in green and red *Amaranthus* species grown in the open field are defense mechanism metabolites (DMM), while allantoin serves as allelochemicals and adenosine, alanine, choline, and aspartate serve as stress-regulating metabolites. Choline in this instance is assumed to serve a dual-purpose role as a stress-regulator and flavour agent. It is assumed that the combination of the released metabolites represents the chemical compounds responsible for influencing effective growth, protecting amaranth against pests/insects and diseases, and serve as self-defense from herbivores. Plants are often attacked by herbivores with a wide range of feeding modes and preferences. Panichikkal and Krishnankutty [55] reported that the presence of organic acids is a unique feature of plant metabolism compared to the metabolisms of animals and microorganisms. Plants accumulate and store organic acids in the cell vacuoles during photosynthesis, which are converted into sugars as food for plants at night and are associated with plant tolerance to environmental stresses [56]. According to Carvalho et al. [57] allelochemicals are defined as beneficial or harmful influence chemical substances that can affect physiological functions as seed germination, respiration, photosynthesis, ion uptake, enzyme activity, water status, transpiration, stomatal opening, and hormone levels. Furthermore, Scognamiglio et al. [58] reported that most allelochemicals are released into the environment by exudation, vaporization, leaching or decomposition.

Although the role of alanine is still not clearly defined, nonetheless, it is linked to being produced as a metabolomic response of plants with oxygen deficiency as reported by Sousa and Sodek [59] which opens a gap for detailed future research.

Plants utilize sunlight as an energy source which acts as an essential environmental signal to regulate growth and developmental processes via photosynthesis [60]. Interestingly, plant sensitivity to radiation varies between plant species and varieties [61,62]. According to Dawood et al. [63] plants possess a plethora of defense strategies to combat the stressors, including UltraViolet C (UVC). A study by Kanani et al. [64] reported that exogenous allantoin might be used to improve UVC stress tolerance due to its ability to regulate plant tolerance to several abiotic stresses such as nutrient deprivation, drought, and salinity. In addition, a study by Dawood et al. [63] found that allantoin increased the epicuticular wax accumulation on the leaf surface of UVC-stressed plants. Furthermore, allantoin-treated plants showed little leaflet-rolling compared to only UVC-stressed plants. During periods of stress, plants have been observed to accumulate allantoin, which is an intermediate compound in the breakdown of purines, as reported by Takagi et al. [65]. Allantoin is essential for plant survival during stressful conditions and plays a significant role in various aspects of plant biology, including metabolism, signaling, bioenergetics, and hereditary processes [66]. A study by Sivelnte et al. [66] reported that allantoin is essential for plant survival during stressful conditions and plays a significant role in various aspects of plant biology including metabolism, signaling, bioenergetics, and hereditary processes.

Notably, the present study makes it the first to detect allantoin (secondary metabolite) in the leaves of *Amaranthus*, which to date has not been researched in detail. In the current study, it is assumed that the synthesis and release of allantoin makes it the main protective metabolite used by *Amaranthus* species grown in open field and greenhouses to defend itself from abiotic and biotic stresses.

Our findings agree with [67] which reported that specific intermediate metabolites such as allantoin could also function as stress-tolerant in plants as a part of nitrogen metabolism and can be considered as a purine alkaloid occurring in vascular plants and lichens [68,69]. In agreement with the aforementioned points, Watanabe et al., Irani, Todd, Werner, and Witte [70,71,72] described allantoin as a nitrogenous compound and considered it a molecule that plays a significant role in mitigating various stress factors. In addition to nitrogen transport and storage in plants, allantoin is reported to be involved in adaptation to environmental stress and lessening of toxic effects of stress factors [73,74,75]. Studies by Dresler et al. [76,77] reported that allantoin significantly reduces the toxic effects of metals by the activation of antioxidant mechanisms in genetically modified plants and through an increase in the level of non-enzymatic antioxidants after exogenous application.

Plants have defense mechanisms such as induced systemic resistance (ISR) and systemic-acquired resistance [4]. In the current study, carnitine and caffeine found in the leaves of *Amaranthus* species grown in the open field are assumed to be a defense mechanism metabolite (DMM). Lescano et al. [78] agree with our findings that carnitine is among the metabolites that play a diverse role in plant defense. Furthermore, Lescano et al. [78] reported that carnitine enhances the tolerance capacity of an organism towards salt stress. In addition, a study by Dresler et al. [79] reported that carnitine made the organism more tolerant to sodium chloride. On the other hand, Jinal et al. [80] indicated that carnitine conjugates are derived from both the catabolism of branched-chain amino acids (CoA) and fatty acid oxidation, and are used to transport fatty acids across membranes and serve as an alternative acyl-group receiver to buffer the CoA pool, typically under stressful conditions when CoA becomes limited [81]. Kets et al. [82] also agree with the above mentioned point that carnitine is used in the transportation of lipid acids within the cell. Plants have developed a variety of defense systems by producing secondary metabolites which directly or indirectly protect the host plant [83,84].

According to Kim et al. [85] caffeine is a major purine alkaloid that has been produced in several species such as tea, cacao, coffee, and kola, and is long known for its role in plant defense [86]. Caffeine has been reported to enable plants to exhibit a strong odour that repels insect and pest (herbivores) attack, virus, and bacterial infection, inhibiting pathogenic growth [87]. In addition, caffeine has been reported to be released upon wounding [87], high-salt, and high-light conditions [87] in order to confer a toxic effect against pathogen infection [85] and prevent herbivore attacks [86], known to affect the growth and development of plants. Furthermore, a study by Ashihara et al. [88] indicated that caffeine is involved in chemical defense and allelopathy.

A study by Guo et al. [89] agrees with our findings that alanine and aspartate are metabolites released by plants to regulate salt stress and alter the movement of heavy metals by acidification [90]. Soil salinization has been reported to reduce the growth and yield of grains [91]. In addition, toxic heavy metals may pose a threat to human beings through the consumption of vegetables [89]. In the current study, it is assumed that green *Amaranthus* species grown in the open field exude aspartate to allow good adaptability to salinity in the soil as compared to other vegetables as indicated by Li et al. [5].

A study by Pinna et al. [92] revealed that choline is released by plants to tolerate salinity stress. In addition, Zeisel and Zhang et al. [93,94] indicated that choline plays a vital role as a key component in osmoregulation, plant stress resistance, and as a food additive. Furthermore, Hernández-Ledesma [95] reported that high content of choline and betaine were found in quinoa, (*Chenopodium quinoa* Willd.) which is able to tolerate extreme environmental conditions (salinity, cold, solar radiation, and drought), and can be cultivated in high altitudes in the mountain areas. From the current study, it is therefore assumed that choline released by green and red *Amaranthus* species play a role as stress regulatory metabolites but might be responsible for flavour and texture on crops that belong to the Amaranthaceae family. Traditionally, *Amaranthus* species are known to exude a distinct aroma as they boil and/or cook which might be attributed to choline. Surprisingly, *Amaranthus* species are not among several food sources such as oil seed crops, animal origin, poultry, vegetables of Brassicaceae family (cabbage, cauliflower, kale, brussels sprouts, Chinese cabbage, broccoli, etc.), onion family (garden onion, Allium), root and shoot vegetables, and leaf vegetables (spinach, jute, lettuce, common beet, swiss chard, pea shoot, etc.) listed on HMBD database [30] reported to be a natural and/or synthetic origin of choline which can be consumed as derivatives to attain choline, which makes the current study the first to report *Amaranthus* species grown in the open field as a source of choline. Choline detected in the current study could be the main attribution to amaranth to be cooked in supplement with a variety of green leafy vegetables such as jute mallow, pumpkin leaves, black nightshade, etc., particularly in the northern part of South Africa (indigenous knowledge and personal observation) as food additives for flavour enhancement and additional texture. It is also important to note that *Amaranthus* is never cooked on its own but in addition to other vegetables as mentioned above.

Furthermore, choline is reported to be a precursor, synthesizer, and enhancer [96] of glycine betaine and a strong Osmo protectant molecule [92]. On the contrary, a study by Nemadodzi et al. [18] disagreed with our findings and reported that choline was the growth-inhibiting metabolite.

Contrary to our findings, a study by Bora, Cai et al. [97,98] reported different flavonoids and phenolic compounds in the leaves of *Amaranthus* which are considered beneficial for human health due to their nutritional and antioxidant properties [99,100,101]. The findings reported by Fasuyi et al. [2] on amino acid percentages, of alanine, and carnitine detected on proximate analysis of *A*. *cruentus* leaf meal agree with the results of the current study. According to Sharma et al. [60] indigenous African leafy vegetables vary enormously in their secondary plant metabolites. In addition, Neugart et al. [102] stated that plants synthesize a diversity of secondary metabolites which protect and repel plants against predators and microbes as per the toxic nature of microbes and herbivores. Some secondary metabolites are reported to help the plant to communicate with other organisms whilst some protect plants from abiotic stress e.g., UV-B radiation [103]. ^1^H NMR based metabolomics study conducted on Lupinus albus or white lupine by Hellal et al. [104] identified caprate, adenosine, and asparagine metabolites in Lupinus albus and were reported to be key compounds that might be responsible for the strong α-glucosidase inhibitory and DPPH radical scavenging activities. Prusinski [105] indicated that previous studies have reported that amino acids have a beneficial effect on the physiological condition of the human organism, especially for those suffering from diabetes. On the other hand, adenosine detected in green and red *Amaranthus* species grown in open field is reported to be induced as a signal and/or stimulus response for wounds/and stress in plants [106]. Our results align with those reported by Khan et al. [107] which says that compounds such as adenosine, alanine, and allantoin, were prominently accumulated in the leaves of plants grown under controlled conditions and during prolonged drought conditions. In addition, Zhao et al. [108] agree with our findings that the release of fatty acids such as caprate from the plant membrane is reported to be involved in plant tolerance to biotic and abiotic stresses. Furthermore, amino acids such as alanine and glutamine were reported in Dill (*Anethum graveolens* L.) grown in open door and greenhouse conditions as reported by Castro-Alves et al. [109], which agrees with our findings on green *Amaranthus* (see Table 1 and Table 2). Surprisingly, in the current study, glutamine was not detected in the leaves of red *Amaranthus* grown in both fields and greenhouses, which warrants future study.

The lack of compounds detected in the leaves of *Amaranthus* species grown in the greenhouse could be attributed to a controlled temperature, regulated water supply, and non-harsh environments in which plants grow effectively without attacks from pests and diseases which could inhibit the release of growth-promoting metabolites, growth-regulating metabolites, and defence mechanism metabolites. According to [16], greenhouses are reported to provide favourable conditions for the plant, thus, growth favourable environments can influence a plant’s composition and performance [110] which could have attributed to low production, synthesis, and release of metabolites in the current study on *Amaranthus* species grown in the greenhouse. The cultivation of crops in greenhouses and/or controlled environments poses fewer production risks, provides protection and shelter to plants in contrast to open-field grown crops, which are exposed to greater environmental changes and depend on natural factors as reported by [16]. Furthermore, greenhouses permit more efficient use of water and inputs and better control of pests, weeds, and diseases [111].

However, in the open field, environmental conditions are unpredictable, unreliable, and inconsistent which may induce plants to react accordingly through the release of pheromones, volatile organic compounds, and plant secondary metabolites to survive the harsh environmental conditions and herbivore attacks. In addition, open field cultivation is subjected to extreme weather such as excessive heat, storms, hail, runoff, and erosion, and is susceptible to pests and diseases.

## 5. Conclusions and Recommendation

Plants naturally synthesize a diverse and wide range of secondary metabolites which help in promoting and/or regulating growth to be used as part of their defence against attacks by herbivorous, insects, and pathogens, and as a means to survive in harsh environment conditions known to slow growth. ^1^H Nuclear magnetic resonance (NMR) is an advanced and efficient metabolomic tool fast-gaining popularity in analyzing and unearthing the biological constituents of indigenous leafy vegetables mostly grown and consumed in developing countries in Africa. Detection of different metabolites such as trehalose, glutamine are reported to serve dual-roles as growth-promoting metabolites and growth-regulating metabolites. Alanine, carnitine, and allantoin have been proven to serve multi-purposes as defence mechanism metabolites against herbivores and pathogens, and stress tolerance against salt accumulation, adverse conditions, water scarcity, and drought. Choline was reported to be the metabolite responsible for flavour and the texture of *Amaranthus* species will help in total comprehension of the impact of cropping systems in the growth and development of *Amaranthus* species. The fluctuation and unpredictable adverse weather conditions in the open field, exposure to herbivores, diseases, and pests, influence the release of volatile organic compounds, pheromones, and/or anti-nutrients against growth-inhibiting factors. The use of ^1^H NMR made the current study the first to detect and report the availability of allantoin and choline in the leaves of green and red Amaranthus. Future studies will include the growing of green and red Amaranthus species, inoculating the crop with the detected metabolites, and comparing it with control plants to confirm the reported roles of metabolites found in the current study.

## Figures and Tables

**Table 1:** NMR chemical shifts (ppm) of metabolites detected on green amaranth grown in the open field cropping system.

Open Field-Grown			
Metabolites	NMR Region	Chenomx 9.0	Human Metabolome Data Base
Carnitine (1)	3.20	2.4	N/A
		3.2	
		3.4	
		4.6	
Caffeine (2)	3.49	3.3	3.22
		3.5	3.34
		3.9	4.04
		7.9	8.49
			Sreekumar et al. [29]
Betaine (3)	3.30	3.3	3.25
	3.9	3.9	3.89
			Wishart et al. [30]
Trehalose (4)	3.41	3.4	3.42
		3.6	3.49
		3.8	3.63
		3.9	3.75
		5.2	3.81
			3.84
			3.85
			4.12
			5.18
			Wishart et al. [30]
Alanine (5)	1.47	1.5	1.47
	1.49	3.8	3.77
Adenosine (6)	4.31	3.8	3.49
		3.9	3.75
		4.3	3.91
		4.4	4.62
		4.8	4.73
		6.1	8.49
		8.2	
		8.3	
Aspartate (7)	2.86	2.7	1.53
	3.90	2.8	1.59
		3.9	1.88
			3.17
			3.77
Glutamine (8)	3.86	2.1	2.11
		2.4	2.42
		2.5	2.46
		3.8	3.76
		6.9	
		7.6	
Caprate (9)	2.10	0.8	N/A
		1.3	
		1.5	
		2.1	
Choline (10)	3.28	3.2	N/A
		3.5	
		4.1	
Sucrose (11)	4.24	3.4	N/A
		3.6	
		3.7	
		3.8	
		3.9	
		4.2	
		4.0	
		5.4	
		Wishart et al. [30]	
Asparagine (12)	6.9	2.8	2.84
		2.9	3.84
		4.0	
		6.9	
		7.6	

**Table 2:** The NMR chemical shifts (ppm) of metabolites detected on green amaranth grown in the greenhouse cropping system.

Greenhouse-Grown			
Metabolites	NMR Region (ppm)	Chenomx	HMDB
Carnitine	3.25	2.4	N/A
		3.2	
		3.44.6	
Allantoin (2)	5.40	5.4	4.120
		6.0	
		7.3	
		8.0	
Glutamine (3)	6.89	2.1	2.11
		2.4	2.42
		2.5	2.46
		3.8	3.76
		6.9	
		7.6	
Trehalose (4)	3.41	3.4	3.42
		3.6	3.49
		3.8	3.63
		3.9	3.75
		5.2	3.81
			3.84
			3.85
			4.12
			5.18
			Wishart et al. [30]
Choline (5)	3.25	3.2	N/A
		3.5	
		4.1	
Caffeine (6)	3.29	3.3	3.22
	3.5	3.5	3.34
	3.91	3.9	4.04
		7.9	8.49
			Sreekumar et al. [29]
Sucrose (7)	4.0	3.4	N/A
	5.38	3.6	
		3.7	
		3.8	
		3.9	
		4.0	
		4.2	
		5.4	
		Wishart et al. [30]	
Betaine (8)	3.25	3.3	3.25
	3.89	3.9	3.89
			Wishart et al. [30]
Alanine (9)	1.45	1.5	1.47
	1.46	3.8	3.77
Asparagine (10)	6.87	2.8	2.84
		2.9	3.4
		4.0	
		6.9	
		7.6	

**Table 3:** NMR chemical shifts (ppm) of metabolites detected on red amaranth grown in open field.

Open Field-Grown			
Metabolite	NMR Region	Chenomx	HMDB
Carnitine (1)	3.21	2.4	N/A
		3.2	
		3.4	
		4.6	
Allantoin (2)	5.35	5.4	4.120
		6.0	
		7.3	
		8.0	
Trehalose (3)	5.23	3.4	3.42
		3.6	3.49
		3.8	3.63
		3.9	3.75
		5.2	3.81
			3.84
			3.85
			4.12
			5.18
			Wishart et al. [30]
Alanine (4)	1.46	1.5	1.47
	1.47	3.8	3.77
Betaine (5)	3.25	3.3	3.25
	3.9	3.9	3.89
			Wishart et al. [30]
Choline (6)	3.19	3.2	N/A
		3.5	
		4.1	
Sucrose (7)	3.49	3.4	N/A
	3.7	3.6	
	4.0	3.7	
		3.8	
		3.9	
		4.0	
		4.2	
		5.4	
		Wishart et al. [30]	

**Table 4:** NMR chemical shifts (ppm) of metabolites detected on red amaranth grown in the greenhouse cultivated.

Greenhouse-Grown			
Metabolite	NMR Region (ppm)	Chenomx	HMDB
carnitine (1)	3.19	2.4	4.120
		3.3	
		3.4	
		4.6	
		6.0	
		7.3	
		8.0	
Allantoin (2)	5.35	5.4	N/A
		6.0	
		7.3	
		8.0	
Caffeine (3)	3.3	3.3	3.22
	3.47	3.5	3.34
	3.91	3.9	4.04
		7.9	8.49
			Sreekumar et al. [29]
Betaine (4)	3.25	3.3	3.25
	3.89	3.9	3.89
			Wishart et al. [30]
Alanine (5)	1.45	1.5	1.47
	1.46	3.8	3.77
Trehalose (6)	3.46	3.4	3.42
	5.16	3.6	3.49
		3.8	3.63
		3.9	3.75
		5.2	3.81
			3.84
			3.85
			4.12
			5.18
			Wishart et al. [30]
Choline (7)	3.19	3.2	N/A
		3.5	
		4.1	
Sucrose (8)	4.21	3.4	N/A
	5.38	3.6	
		3.7	
		3.8	
		3.9	
		4.0	
		4.2	
		5.4	
		Wishart et al. [30]	

## Data Availability

The data presented in this study are available on request from the corresponding author. The data are not publicly available due to privacy.

## References

[1] Liu F., Stutzel H. (2004). Biomass partitioning, specific leaf area, and water use efficiency of vegetable amaranth (*Amaranthus* spp.) in response to drought stress. Sci. Hortic..

[2] Fasuyi A.O., Dairo F.A.S., Adeniji A.O. (2008). Tropical vegetable (*Amaranthus cruentus*) leaf meal as alternative protein supplement in broiler starter diets: Bionutritional evaluation. J. Cent. Eur. Agric..

[3] Pisarikova B., Zraly Z., Kracmar S., Trckova M., Herzig I. (2006). The use of amaranth (genus *Amaranthus* L.) in the diets for broiler chickens. Vet. Med..

[4] Sauer J.D. (1993). Amaranthaceae: Amaranth family. Historical Geography of Crop Plants: A Select Roster.

[5] Li Q., Cai S., Mo C., Chu B., Peng L., Yang F. (2010). Toxic effects of heavy metals and their accumulation in vegetables grown in a saline soil. Ecotoxicol. Environ. Saf..

[6] Sogbohossou O.E.D., Achigan-Dako E.G. (2014). Phenetic differentiation and use-type delimitation in *Amaranthus* spp. from worldwide origins. Sci. Hortic..

[7] Trucco F., Tranel P.J., Kole C. (2011). Amaranthus. Wild Crop Relatives: Genomic and Breeding Resources, Vegetable.

[8] Dhillon G.S., Kaur S., Brar S.K. (2013). Perspective of apple processing wastes as low-cost substrates for bioproduction of high value products: A review. Renew. Sust. Energ. Rev..

[9] Ramos-Diaz J.M., Kirjoranta S., Tenitz S., Penttila P.A., Serimaa R., Lampi A.M., Jouppila K.K. (2013). Use of amaranth, quinoa and kaniwa in extruded corn-based snacks. J. Cereal. Sci..

[10] Mallory M.A., Hall R.V., McNabb A.R., Pratt D.B., Jellen E.N., Maughan P.J. (2008). Development and characterization of microsatellite markers for the grain amaranths. Crop. Sci..

[11] Managa G.M., Nemadodzi L.E. (2023). Comparison of agronomic parameters and nutritional composition of red and green amaranth species grown in open field versus greenhouse environment. Agriculture.

[12] Li H., Deng Z., Liu R., Zhu H., Draves J., Marcone M., Sun Y., Tsao R. (2015). Characterization of phenolics, betacyanins and antioxidant activities of the seed, leaf, sprout, flower and stalk extracts of three *Amaranthus* species. J. Food Compos. Anal..

[13] Sarkar R., Nandan C.K., Mandal S., Patra P., Das D., Islam S.S. (2009). Structural characterization of a heteropolysaccharide isolated from hot water extract of the stems of *Amaranthus tricolor* Linn. (*Amaranthus gangeticus* L.). Carbohydr. Res..

[14] Ishtiaq S., Ahmad M., Hanif U., Akbar S., Kamran S.H. (2014). Phytochemical and in vitro antioxidant evaluation of different fractions of *Amaranthus graecizans* subsp *silvestris* (Vill.) Brenan. Asian Pac. J. Trop. Med..

[15] Venskutonis P.R., Kraujalis P. (2013). Nutritional components of amaranth seeds and vegetables: A Review on composition, properties and uses. Comp. Rev. Food Sci. Food Saf..

[16] Chávez-Servín J.L., Cabrera-Baeza H.F., Jiménez Ugalde E.A., Mercado-Luna A., de la Torre-Carbot K., Escobar-García K., Barreyro A.A., Serrano-Arellano J., García-Gasca T. (2017). Comparison of chemical composition and growth of amaranth (*Amaranthus hypochondriacus*) between greenhouse and open field Systems. Int. J. Agric. Biol..

[17] Fuller R., Zahnd A. (2012). Solar greenhouse technology for food security: A case study from Humla District, NW Nepal. Mt. Res. Dev..

[18] Nemadodzi L.E., Vervoort J., Prinsloo G. (2020). NMR-Based Metabolomic Analysis and Microbial Composition of Soil Supporting *Burkea africana* Growth. Metabolites.

[19] Anand A., Sharma A., Kaur Saini H., Sharma S., Sharma R., Thakur C., Atanassova M., Caruso G., Pasdaran A. (2022). Profiling of Plant Derived Natural Constituents by Using Magnetic Resonance Techniques. Concepts Magn. Reson. Part A Bridg. Educ. Res..

[20] Altemimi A., Lakhssassi N., Baharlouei D., Watson A., Lightfoot D. (2017). Phytochemicals: Extraction, isolation, and identification of bioactive compounds from plant extracts. Plants.

[21] Yeo H.J., Baek S.A., Sathasivam R., Kim J.K., Park S.U. (2021). Metabolomic analysis reveals the interaction of primary and secondary metabolism in white, pale green, and green pak choi (*Brassica rapa* subsp. *chinensis*). Appl. Biol. Chem..

[22] Zandalinas S.I., Mittler R., Balfagón D., Arbona V., Gómez-Cadenas A. (2018). Plant adaptations to the combination of drought and high temperatures. Physiol. Plant..

[23] Nicholson J., Lindon J., Holmes E. (1999). “Metabolomics”: Understanding the metabolomics response of living systems to pathophysiological stimuli via multivariate statistical analysis of biological NMR spectroscopic data. Xenobiotica.

[24] Mthimunye L.M., Managa G.M., Nemadodzi L.E. (2023). The Influence of Lablab Purpureus Growth on Nitrogen Availability and Mineral Composition Concentration in Nutrient Poor Savanna Soils. Agron J..

[25] Kim H.K., Choi Y.H., Verpoorte R. (2010). NMR-based metabolomic analysis of plants. Nature.

[26] Maree J., Viljoen A. (2012). Phytochemical distinction between *Pelargonium sidoides* and *Pelargonium reniforme*—A quality control perspective. S. Afr. J. Bot..

[27] Fernie A.R., Aharoni A., Wilmitzer L., Stutt M., Tohge T., Kopka J., Carol A.J., Saito K., Fraser P.D., Deluca V. (2011). Recommendations for reporting metabolite data. Plant Cell.

[28] Mediani A., Abas F., Khatib A., Maulidiani M., Shaari K., Choi Y.H., Lajis N. (2012). 1H-NMR-based metabolomics approach to understanding the drying e_ects on the phytochemicals in *Cosmos caudatus*. Food Res. Int..

[29] Sreekumar A., Poisson L.M., Rajendiran T.M., Khan A.P., Cao Q., Yu J., Laxman B., Mehra R., Lonigro R.J., Li Y. (2009). Metabolomic profiles delineate potential role for sarcosine in prostate cancer progression. Nature.

[30] Wishart D.S., Knox C., Guo A.C., Eisner R., Young N., Gautam B., Hau D.D., Psychogios N., Dong E., Bouatra S. (2009). HMDB: A knowledgebase for the human metabolome. Nucleic Acids Res..

[31] Modi M., Modi A., Hendriks S. (2006). Potential role for wild vegetables in household food security: A preliminary case study in Kwazulu-Natal, South Africa. Afr. J. Food Agric. Nutr. Dev..

[32] Van Rensburg W.J., Venter S.L., Netshiluvhi T.R., Van Den Heever E., Vorster H.J., De Ronde J.A., Bornman C.H. (2004). Role of indigenous leafy vegetables in combating hunger and malnutrition. S. Afr. J. Bot..

[33] Sunita K., Mishra I., Mishra J., Prakash J., Arora N.K. (2020). Secondary Metabolites from Halotolerant Plant Growth Promoting Rhizobacteria for Ameliorating Salinity Stress in Plants. Front. Microbiol..

[34] Nadeem S.M., Zahir Z.A., Naveed M., Nawaz S. (2013). Mitigation of salinity-induced negative impact on the growth and yield of wheat by plant growth-promoting rhizobacteria in naturally saline conditions. Ann. Microbiol..

[35] Eastmond P.J., van Dijken A.J.H., Spielman M., Kerr A., Tissier A.F., Dickinson H.G., Jones J.D., Smeekens S.C., Graham I.A. (2002). Trehalose-6-phosphate synthase 1, which catalyses the first step in trehalose synthesis, is essential for *Arabidopsis embryo* maturation. Plant J..

[36] Dijken A.J.V., Schluepmann H., Smeekens S.C. (2004). Arabidopsis trehalose-6-phosphate synthase 1 is essential for normal vegetative growth and transition to flowering. Plant Physiol..

[37] Thierry L., Delatte2 P.S., Youichi K., Minami M., Gerhardus J., de Jong G.W., Somsen A., Wiese-Klinkenberg L.F.P., Matthew J.P., Henriette S. (2011). Growth Arrest by Trehalose-6-Phosphate: An Astonishing Case of Primary Metabolite Control overgrowth by Way of the SnRK1 Signaling Pathway. Plant Physiol..

[38] Schluepmann H., Paul M. (2009). Trehalose Metabolites in Arabidopsis—Elusive, active and central. Arab. Book.

[39] Tisarum R., Theerawitaya C., Samphumphuang T., Singh H.P., Cha-um S. (2020). Foliar application of glycinebetaine regulates soluble sugars and modulates physiological adaptations in sweet potato (*Ipomoea batatas*) under water deficit. Protoplasma.

[40] Naidu B.P., Cameron D.F., Konduri S.V. Improving drought tolerance of cotton by glycine betaine application and selection. Proceedings of the Australian Agronomy Conference.

[41] Rahman M.S., Miyake H., Takeoka Y. (2002). Effects of exogenous glycine betaine on growth and ultrastructure of salt-stressed rice seedlings (*Oryza sativa* L.). Plant Prod. Sci..

[42] Hwang I.S., An S.H., Hwang B.K. (2011). Pepper asparagine synthetase 1 (CaAS1) is required for plant nitrogen assimilation and defense responses to microbial pathogens. Plant J..

[43] Luo L., Qin R., Liu T., Yu M., Yang T., Xu G. (2018). OsASN1 plays a critical role in asparagine-dependent rice development. Int. J. Mol. Sci..

[44] Gaufichon L., Reisdorf-Cren M., Rothstein S.J., Chardon F., Suzuki A. (2010). Biological functions of asparagine synthetase in plants. Plant Sci..

[45] Ahmad I. (2015). New Insights into Plant Amino Acid Transport and Its Contribution to Nitrogen Nutrition.

[46] Lea P.J., Sodek L., Parry M.A.J., Shewry R., Halford N.G. (2007). Asparagine in plants. Ann. Appl. Biol..

[47] Hayashi H., Chino M. (1990). Chemical-Composition of Phloem Sap from the Uppermost Internode of the Rice Plant. Plant Cell Physiol..

[48] Maluleke M.K. (2022). Metabolite profile of African horned cucumber (*Cucumis metuliferus* E. May. Ex Naudin) fruit grown under differing environmental conditions. Nature.

[49] Nkobole N., Prinsloo G. (2021). 1H-NMR and LC-MS Based Metabolomics Analysis of Wild and Cultivated *Amaranthus* spp.. Molecules.

[50] Ashraf M.A., Iqbal M., Rasheed R., Hussain I., Riaz M., Arif M.S. (2018). Environmental stress and secondary metabolites in plants: An overview. Plant Metab. Regul. Environ. Stress.

[51] Federal G. (1988). Plant mechanical defenses against insect herbivory. Biologia.

[52] Chua L.S. (2016). Untargeted MS-based small metabolite identification from the plant leaves and stems of *Impatiens balsamina*. Plant Physiol. Biochem..

[53] Lopez-Bucio J., Nieto-Jacobo M.F., Ramırez-Rodrıguez V., Herrera-Estrella L. (2000). Organic acid metabolism in plants: From adaptive physiology to transgenic varieties for cultivation in extreme soils. Plant Sci..

[54] Obata T., Witt S., Lisec J., Palacios-Rojas N., Florez-Sarasa I., Yousfi S., Araus J.L., Cairns J.E., Fernie A.R. (2015). Metabolite profiles of maize leaves in drought, heat, and combined stress field trials reveal the relationship between metabolism and grain yield. Plant Physiol..

[55] Panichikkal J., Krishnankutty R.E. (2021). Root exudate components induced production of plant beneficial metabolites in rhizospheric *Pseudomonas* spp.. Rhizosphere.

[56] Wishart D.S., Guo A., Oler E., Wang F., Anjum A., Peters H., Dizon R., Sayeeda Z., Tian S., Lee B.L. (2022). HMDB 5.0: The human metabolome database for. Nucleic Acids Res..

[57] Carvalho M.S.S., Andrade-Vieira L.F., dos Santos F.E., Correa F.F., das Graças Cardoso M., Vilela L.R. (2019). Allelopathic potential and phytochemical screening of ethanolic extracts from five species of *Amaranthus* spp. in the plant model *Lactuca sativa*. Sci. Hortic..

[58] Scognamiglio M., D’Abrosca B., Esposito A., Pacifico S., Monaco P., Fiorentino A. (2013). Plant growth inhibitors: Allelopathic role or phytotoxic effects? Focus on Mediterranean biomes. Phytochem. Rev..

[59] Sousa C.A.F.D., Sodek L. (2002). The metabolic response of plants to oxygen deficiency. Braz. J. Plant Physiol..

[60] Sharma A., Kumar V., Shahzad B., Ramakrishnan M., Singh Sidhu G.P., Bali A.S., Handa N., Kapoor D., Yadav P., Khanna K. (2020). Photosynthetic response of plants under different abiotic stresses: A review. J. Plant Growth Regul..

[61] Correia C.M., Areal E.L.V., Torres-Pereira M.S., Torres-Pereira J.M.G. (1999). Intraspecific variation in sensitivity to ultraviolet-B radiation in maize grown under field conditions: II. Physiological and biochemical aspects. Field Crops Res..

[62] Verdaguer D., Jansen M.A.K., Llorens L., Morales L.O., Neugart S. (2017). UV-A radiation effects on higher plants: Exploring the known unknown. Plant Sci..

[63] Dawood M.F., Tahjib-Ul-Arif M., Sohag A.A.M., Abdel Latef A.A.H., Ragaey M.M. (2020). Mechanistic insight of allantoin in protecting tomato plants against ultraviolet c stress. Plants.

[64] Kanani H., Dutta B., Klapa M.I. (2010). Individual vs. combinatorial effect of elevated CO_2_ conditions and salinity stress on *Arabidopsis thaliana* liquid cultures: Comparing the early molecular response using time-series transcriptomic and metabolomic analyses. BMC Syst. Biol..

[65] Takagi H., Ishiga Y., Watanabe S., Konishi T., Egusa M., Akiyoshi N., Shimada H. (2016). Allantoin, a stress-related purine metabolite, canactivate jasmonate signaling in a MYC2-regulated and abscisic acid-dependent manner. J. Exp. Bot..

[66] Silvente S., Sobolev A.P., Lara M. (2012). Metabolite adjustments in drought tolerant and sensitive soybean genotypes in response to waterstress. PLoS ONE.

[67] Alamillo J.M., DÍAz-Leal J.L., SÁNchez-Moran M.V., Pineda M. (2010). Molecular analysis of ureide accumulation under drought stress in *Phaseolus vulgaris* L.. Plant Cell Environ..

[68] Rose M.T., Rose T.J., Pariasca-Tanaka J., Yoshihashi T., Neuweger H., Goesmann A., Frei M., Wissuwa M. (2012). Root metabolic response of rice (*Oryza sativa* L.) genotypes with contrasting tolerance to zinc deficiency and bicarbonate excess. Planta.

[69] Yobi A., Wone B.W.M., Xu W., Alexander D.C., Guo L., Ryals J.A., Oliver M.J., Cushman J.C. (2013). Metabolomic profiling in *Selaginella lepidophylla* at various hydration states provides new insights into the mechanistic basis of desiccation tolerance. Mol. Plant..

[70] Watanabe S., Matsumoto M., Hakomori Y., Takagi H., Shimada H., Sakamoto A. (2014). The purine metabolite allantoin enhances abiotic stress tolerance through synergistic activation of abscisic acid metabolism. Plant Cell Environ..

[71] Irani S., Todd C.D. (2018). Exogenous allantoin increases Arabidopsis seedlings tolerance to NaCl stress and regulates expression of oxidative stress response genes. J. Plant Physiol..

[72] Werner A.K., Witte C.-P. (2011). The biochemistry of nitrogen mobilization: Purine ring catabolism. Trends Plant Sci..

[73] Dresler S., Rutkowska E., Bednarek W., Stanisławski G., Kubrak T., Bogucka-Kocka A., Wójcik M. (2017). Selected secondary metabolites in *Echium vulgare* L. populations from nonmetalliferous and metalliferous areas. Phytochemistry.

[74] Dresler S., Kováčik J., Wójciak H., Sowa I., Strzemski M., Wójciak M. (2021). Allantoin content in lichens depends on anthropopressure level. Ecol. Indic..

[75] Nourimand M., Todd C.D. (2016). Allantoin increases cadmium tolerance in Arabidopsis via activation of antioxidant mechanisms. Plant Cell Physiol..

[76] Dresler S., Szymczak G., Wójcik M. (2017). Comparison of some secondary metabolite content in the seventeen species of the Boraginaceae family. Pharm. Biol..

[77] Dresler S., Hawrylak-Nowak B., Kováčik J., Woźniak M., Gałązka A., Staniak M., Wójciak M., Sowa I. (2021). Organic nitrogen modulates not only cadmium toxicity but also microbial activity in plants. J. Hazard. Mater..

[78] Lescano C.I., Martini C., González C.A., Desimone M. (2016). Allantoin accumulation mediated by allantoinase down regulation and transport by Ureide Permease 5 confers salt stress tolerance to Arabidopsis plants. Plant Mol. Biol..

[79] Dresler S., Hawrylak-Nowak B., Kováčik J., Pochwatka M., Hanaka A., Strzemski M., Sowa I., Wójciak-Kosior I.M. (2019). Allantoin attenuates cadmium-induced toxicity in cucumber plants. Ecotoxicol. Environ. Saf..

[80] Jinal H.N., Sakthivel K., van Leeuwenhoek N.A.A. (2020). Characterisation of antagonistic *Bacillus paralicheniformis* (strain EAL) by LC–MS, antimicrobial peptide genes, and ISR determinants. Antonie Van Leeuwenhoek.

[81] Oney-Birol S. (2019). exogenous L-carnitine promotes plant Growth and cell Division by Mitigating Genotoxic Damage of Salt Stress. Sci. Rep..

[82] Kets E.P.W., Galinski E.A., de Bont J.A.M. (1994). Carnitine: A novel compatible solute in *Lactobacillus plantarum*. Arch Microbiol..

[83] Dixon R.A. (2001). Natural products and plant resistance. Nature.

[84] Dangl J.L., Jones J.D.G. (2001). Plant pathogens and integrated defence response to infection. Nature.

[85] Kim Y.S., Choi Y.E., Sano H. (2010). Plant vaccination: Stimulation of defense system by caffeine production in planta. Plant Signal. Behav..

[86] Li X., Ahammed G.J., Li Z., Tang M., Yan P., Han W. (2016). Decreased biosynthesis of jasmonic acid via lipoxygenase pathway compromised caffeine-induced resistance to *Colletotrichum gloeosporioides* under elevated CO_2_ in tea seedlings. Phytopathology.

[87] Aneja M., Gianfagna T. (2001). Induction and accumulation of caffeine in young, actively growing young leaves of cocoa (*Theobroma cacao* L.) by wounding or infection with *Crinipellis pernisiosa*. Physiol. Mol. Plant. Pathol..

[88] Ashihara H., Sano H., Crozier A. (2008). Caffeine and related purine alkaloids: Biosynthesis, catabolism, function and generic engineering. Phytochemistry.

[89] Guo S.H., Hu N., Li Q.S., Yang P., Wang L.L., Xu Z.M., Chen H.J., He B.Y., Zeng E.Y. (2018). Response of edible amaranth cultivar to salt stress led to Cd mobilization in rhizosphere soil: A metabolomic analysis. Environ. Pollut..

[90] Li T., Liang C., Han X., Yang X. (2013). Mobilization of cadmium by dissolved organic matter in the rhizosphere of hyperaccumulator *Sedum Alfredii*. Chemosphere.

[91] Wang Y. (2013). Land exploitation resulting in soil salinization in a desert–oasis ecotone. Catena.

[92] Pinna M.C., Bauduin P., Touraud D., Monduzzi M., Ninham B.W., Kunz W. (2005). Hofmeister effects in biology: Effect of choline addition on the salt-induced super activity of horseradish peroxidase and its implication for salt resistance of plants. J. Phys. Chem. B.

[93] Zeisel S.H. (2006). Choline: Critical role during fetal development and dietary requirements in adults. Annu. Rev. Nutr..

[94] Zhang H., Murzello C., Sun Y., Kim M.S., Xie X., Jeter R.M., Zak J.C., Dowd S.E., Paré P.W. (2010). Choline and osmotic-stress tolerance induced in Arabidopsis by the soil microbe Bacillus subtilis (GB03). Mol. Plant-Microbe Interact..

[95] Hernández-Ledesma B. (2019). Quinoa (*Chenopodium quinoa* Willd.) as source of bioactive compounds: A review. Bioact. Compd. Health Dis..

[96] Eghtesadi N., Olaifa K., Perna F.M., Capriati V., Trotta M., Ajunwa O., Marsili E. (2022). Electroactivity of weak electricigen Bacillus subtilis biofilms in solution containing deep eutectic solvent components. Bioelectrochemistry.

[97] Bora P. (2014). Anti-Nutritional Factors in Foods and their Effects. J. Acad. Ind. Res..

[98] Cai Y.Z., Sun M., Corke H. (2005). Characterization and application of betalain pigments from plants of the Amaranthaceae: Pigments in food. Trends Food Sci. Tech..

[99] Suryavanshi V.L., Sathe P.A., Baing M.M., Singh G.R., Lakshmi S.N. (2007). Determination of rutin in *Amaranthus spinosus* Linn whole plant powder by HPTLC. Chromatography.

[100] Ferguson G.M., Hamill A.S., Tardif F.J. (2001). ALS inhibitor resistance in populations of *Powell amaranth* and redroot pigweed. Weed Sci..

[101] Srinivasan K., Natarajan D., Dheen M., Perumal G., Mohanasundari C., Prabakar K., Sengottuvel R. (2006). Antibacterial activity of selected medicinal plants. Hamdard Med..

[102] Neugart S., Baldermann S., Ngwene B., Wesonga J., Schreiner M. (2017). Indigenous leafy vegetables of Eastern Africa—A source of extraordinary secondary plant metabolites. Food Res. Int..

[103] Zaynab M., Fatima M., Abbas S., Sharif Y., Umair M., Zafar M.H., Bahadar K. (2018). Role of secondary metabolites in plant defense against pathogens. Microb. Pathog..

[104] Hellal K., Mediani A., Ismail I.S., Tan C.P., Abas F. (2021). 1H NMR-based metabolomics and UHPLC-ESI-MS/MS for the investigation of bioactive compounds from *Lupinus albus* fractions. Food Res. Int..

[105] Prusinski J. (2017). White lupin (*Lupinus albus* L.)–nutritional and health values in human nutrition—A review. Czech J. Food Sci..

[106] Jeter C.R., Tang W., Henaff E., Butterfield T., Roux S.J. (2004). Evidence of a novel cell signaling role for extracellular adenosine triphosphates and diphosphates in Arabidopsis. Plant Cell.

[107] Khan N., Bano A., Rahman M.A., Rathinasabapathi B., Babar M.A. (2019). UPLC-HRMS-based untargeted metabolic profiling reveals changes in chickpea (*Cicer arietinum*) metabolome following long-term drought stress. Plant Cell Environ..

[108] Zhao L., Huang Y., Hu J., Zhou H., Adeleye A.S., Keller A.A. (2016). 1H NMR and GC-MS based metabolomics reveal defense and detoxification mechanism of cucumber plant under nano-Cu stress. Environ. Sci. Technol..

[109] Castro-Alves V., Kalbina I., Nilsen A., Aronsson M., Rosenqvist E., Jansen M.A., Qian M., Öström Å., Hyötyläinen T., Strid Å. (2021). Integration of non-target metabolomics and sensory analysis unravels vegetable plant metabolite signatures associated with sensory quality: A case study using dill (*Anethum graveolens*). Food Chem..

[110] Wilkinson J., Rocha R. (2009). Agro-industry trends, patterns and development impacts. Agro Industries for Development.

[111] Davies P.A. (2005). A solar cooling system for greenhouse food production in hot climates. Sol. Energy.

